# Oxylipin Profiles as Functional Characteristics of Acute Inflammatory Responses in Astrocytes Pre-Treated with IL-4, IL-10, or LPS

**DOI:** 10.3390/ijms21051780

**Published:** 2020-03-05

**Authors:** Dmitry V. Chistyakov, Gleb E. Gavrish, Sergei V. Goriainov, Viktor V. Chistyakov, Alina A. Astakhova, Nadezda V. Azbukina, Marina G. Sergeeva

**Affiliations:** 1 Belozersky Institute of Physico-Chemical Biology, Lomonosov Moscow State University, 119992 Moscow, Russia; alina.an.astakhova@gmail.com (A.A.A.); mg.sergeeva@gmail.com (M.G.S.); 2 Faculty of Bioengineering and Bioinformatics, Lomonosov Moscow State University, 119234 Moscow, Russia; gavrishglebevg@gmail.com (G.E.G.); ridernadya@gmail.com (N.V.A.); 3 SREC PFUR Peoples’ Friendship University of Russia (RUDN University), 117198 Moscow, Russia; goryainovs@list.ru (S.V.G.); chistvic@gmail.com (V.V.C.)

**Keywords:** rat astrocytes, oxylipins, eicosanoids, polarization, interleukins IL-4, IL-10, endotoxin tolerance, inflammation, LPS

## Abstract

Functional phenotypes, which cells can acquire depending on the microenvironment, are currently the focus of investigations into new anti-inflammatory therapeutic approaches. Glial cells, microglia, and astrocytes are major participants in neuroinflammation, but their roles differ, as microglia are cells of mesodermal origin, while astrocytes are cells of ectodermal origin. The inflammatory phenotype of cells can be modulated by ω-6- and ω-3-polyunsaturated fatty acid-derived oxylipins, although data on changes in oxylipin profiles in different cell adaptations to pro- and anti-inflammatory stimuli are scarce. Our study aimed to compare UPLC-MS/MS-measured oxylipin profiles in various rat astrocyte adaptation states. We used cells treated for 24 h with lipopolysaccharide (LPS) for classical pro-inflammatory adaptation and with interleukin 4 (IL-4) or 10 (IL-10) for alternative anti-inflammatory adaptation, with the resulting phenotypes characterized by quantitative real-time PCR (RT-PCR). We also tested long-term, low-concentration LPS treatment (endotoxin treatment) as a model of astrocyte adaptations. The functional response of astrocytes was estimated by acute (4 h) LPS-induced cell reactivity, measured by gene expression markers and oxylipin synthesis. We discovered that, as well as gene markers, oxylipin profiles can serve as markers of pro- (A1-like) or anti-inflammatory (A2-like) adaptations. We observed predominant involvement of ω-6 polyunsaturated fatty acid (PUFA) and the cyclooxygenase branch for classical (LPS) pro-inflammatory adaptations and ω-3 PUFA and the lipoxygenase branch for alternative (IL-4) anti-inflammatory adaptations. Treatment with IL-4, but not IL-10, primes the ability of astrocytes to activate the innate immunity signaling pathways in response to LPS. Endotoxin-treated astrocytes provide an alternative anti-inflammatory adaptation, which makes cells less sensitive to acute LPS stimulation than the IL-4 induced adaptation. Taken together, the data reveal that oxylipin profiles associate with different states of polarization to generate a pro-inflammatory or anti-inflammatory phenotype. This association manifests itself both in native cells and in their responses to a pro-inflammatory stimulus.

## 1. Introduction

The notion that cells can acquire distinct functional phenotypes depending on the microenvironment is now supported by a large body of data. In the case of myeloid cells, two well-known polarized phenotypes are referred to as classically activated macrophages (M1 macrophages) and alternatively activated macrophages (M2 macrophages) [1]. These phenotypes are induced by multiple factors and are characterized by the expression of transcriptional modules that underlie specialized functions [2]. Research in this area, together with data on the resolution of inflammatory mechanisms [3], open up new directions in the search for ways to normalize inflammatory processes. Although the polarization phenomenon can be attributed to a particular state of cells [1,2], the question of how adaptation to the microenvironment (for example, the action of pro- or anti-inflammatory stimuli over a long duration) can change the responses of cells to pro-inflammatory stimuli remains open. It is important to note that, although the involvement of oxidized derivatives of ω-3 and ω-6 polyunsaturated fatty acids (PUFAs), especially prostaglandins (PGs) and leukotrienes, in an inflammatory response has been known for a long time (rev. in. [4]), their involvement as substances for resolution is a relatively novel research aspect [3]. The conversion of PUFAs into oxylipins occurs via three major pathways, involving cyclooxygenases (COX), lipoxygenases (LOX), and cytochrome P450 monooxygenases (CYP450) [5]. This class of substances includes both pro-inflammatory compounds and resolution substances, which are responsible for restoring the system after the pro-inflammatory stimulus has been applied [3,6]. There are limited data on the relationship between oxylipins and various cell polarization and/or adaptation states. Recently, it was shown that various ω-3/ω-6 PUFA diets are able to change the polarization of macrophages [7,8]. There is also evidence that ω-6- and ω-3-derived oxylipins are able to modulate the inflammatory phenotype of immune cells, especially macrophages [7,9]. However, data on the changes in oxylipin profiles are scarce.

The activation of glial cells accompanies all disorders of the central nervous system (CNS) related to homeostasis and is an integral destructive factor in such neuropathologies as Alzheimer’s disease, Parkinson’s disease, and multiple sclerosis [10,11,12]. Such plural roles are attributed to the involvement of glial cells, primarily astrocytes and microglia, in immune and inflammatory responses in the CNS [11,12]. Microglia belong to cell types of mesodermal origin and therefore reveal common features in response to inflammatory stimuli as cells of the immune system, monocytes, and macrophages [13]. The presence of M1/M2 phenotypic polarization has also been suggested for microglia [14]. Astrocytes are cells of ectodermal origin and have some specificity in their responses to inflammatory stimuli [15,16,17,18]. These cells fulfill an important role as innate immune cells in the brain [19]. As a regulator of brain inflammation, astrocytes can release various immune and inflammatory mediators, such as pro- and anti-inflammatory cytokines/chemokines and oxylipins, which may subsequently exert neurotoxic or neuroprotective effects [6,12,15]. Astrocytes sense lipopolysaccharide (LPS) signals via the signaling system of Toll-like receptors (TLRs) and promptly respond to pro-inflammatory challenges posed by the activation of downstream signaling cascades, including conventional markers of inflammation: interleukin 6 (IL-6), Tumor necrosis factor alpha (TNFα), COX-2, and CC chemokines [11,20,21]. Key cellular and molecular mechanisms driving the functional implications of different forms of astrocyte reactivity in CNS disorders remain unidentified in neurobiology studies [22].

Under treatments similar to the M1 and M2 polarization of macrophages, astrocytes can acquire various states of phenotypic polarization, named A1 and A2, by analogy [23,24,25]. Mechanisms of these changes and the possibilities of directed regulation are unexplored. Although we characterized astrocyte oxylipin synthesis for pro-inflammatory stimuli [20,26,27], the data concerning oxylipin synthesis in various astrocyte polarization states are missing. Therefore, we stimulated astrocytes with interleukin 4 (IL-4), interleukin 10 (IL-10), and LPS for 24 h (h) and subdivided their phenotypic polarization into A1-like (pro-inflammatory, classical) and A2-like (anti-inflammatory, alternative) using the previously suggested markers [23,28]. Oxylipin synthesis for IL-4-, IL-10-, and LPS-induced polarization states were then evaluated and compared with the data for the so-called endotoxin tolerance model. This is an interesting model for the following reasons. First of all, the endotoxin might contribute to neurodegeneration (rev. in [10]). In addition, for cells of myeloid origin, it is known that the treatment of cells with low concentrations of LPS can reprogram them, such that their response to further acute endotoxin challenges is compromised. The molecular mechanisms underlying endotoxin tolerance remain elusive [10,29]. This prompts an additional question concerning the endotoxin tolerance phenomenon in relation to astrocytes. Recently, we have shown that the phenomenon exists in astrocytes [26]. Accordingly, we included the endotoxin tolerance model in a comparison series of various treatments leading to the adaptation of astrocytes in their ability to respond to acute LPS stimulation. The sensitivity of treated astrocytes to acute stimulation by LPS was estimated by inflammatory marker expression profiles and oxylipin synthesis. The experiments revealed that astrocytes’ adaptations to various microenvironments, modulated by pro- and anti-inflammatory stimuli, changed the oxylipin profiles, which may be relevant to the innate immune responses of these cells.

## 2. Results

### 2.1. Classical or Alternative Activation of Astrocytes in Culture

**Figure 1:**
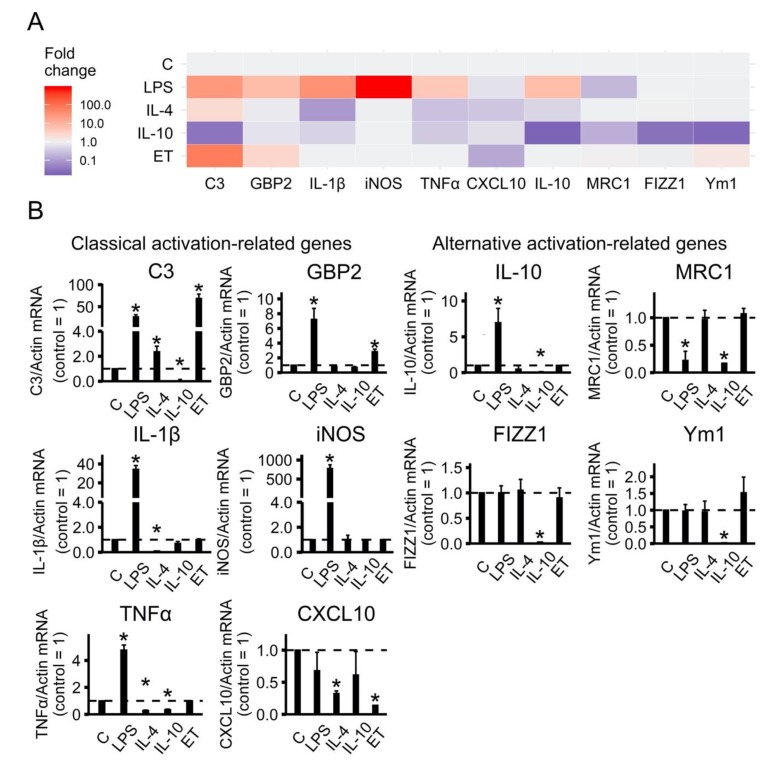
Identification of astrocytes polarization state in primary rat astrocyte cultures based on gene-expression profiles. (**A**) Heat map representation; (**B**) quantitative analysis of expression data. The primary rat astrocyte cultures were treated with lipopolysaccharide (LPS, 100 ng/mL), interleukin 4 (IL-4, 10 ng/mL) or interleukin10 (IL-10, 20 ng/mL) for 24 h or adapted to endotoxin in the tolerance model (ET, LPS 10 ng/mL, 48h), and the total RNAs were isolated. The mRNA levels of polarization state markers (*C3, GBP2, IL-1β, iNOS, TNFα, CXCL10, IL-10, MRC1, FIZZ1* and *Ym1*) were determined by quantitative real-time PCR (RT-PCR). The values are normalized to *β-actin* mRNA levels. The results are expressed as fold-changes, relative to untreated cells. The values represent a mean ± SEM from three independent experiments. * *p* < 0.05, compared with the unstimulated cells.

To characterize whether cultured astrocytes exhibit functionally adaptation states, we estimated the expression profiles of marker genes in rat astrocyte cultures following exposure to LPS (100 ng/mL), IL-4 (10 ng/mL), or IL-10 (20 ng/mL) for 24 h (Figure 1). We also tested whether long-term, low-concentration LPS treatment shifts astrocytes in the direction of an alternative adaptation states. For this, we treated cells with low-concentration LPS (10 ng/mL) for 48 h and tested the mRNA expression of classical and alternative activation-related genes (Figure 1). From now on, we will refer to low-concentration LPS treatment as ET (endotoxin) treatment, to distinguish it from acute stimulation with medium-LPS concentrations. After the cultured astrocytes were treated with the stimuli, quantitative real-time PCR (RT-PCR) analysis was performed to determine the mRNA levels of genes previously associated with either classical (*IL-1β, iNOS, TNFα, C3, GBP2, CXCL10*) or alternative (*IL-10, MRC1, FIZZ1, Ym1*) activations [23,28].

A representative heat map indicates that classical and alternative activation-related genes were observed (Figure 1A). Quantitative analysis revealed that LPS induced classical activation, whereas IL-4 and IL-10 induced two subsets of alternative activation status (Figure 1B). Indeed, LPS stimulation strongly activated most of the pro-inflammatory genes (*C3, GBP2, IL-1β, iNOS, TNFα*) and the anti-inflammatory gene IL-10, but it did not affect the expression of *FIZZ1, Ym1*, or the pro-inflammatory gene *CXCL10* and even led to a decrease in the alternative marker gene MRC1 (Figure 1B). In contrast, alternative activation stimuli (IL-4 or IL-10) did not have a significant effect on pro-inflammatory genes or even slightly decrease them in comparison to the control levels of expression (Figure 1B). It is interesting that on *C3*, which is an important marker of inflammation [30], prolonged treatment of IL-4 leads to the increased expression and treatment of IL-10 to a reduced expression relative to the untreated cells. Moreover, IL-4 did not modulate the examined alternative activation markers, whereas IL-10 treatment has the opposite effect to LPS for IL-10 expression, which reduces expression *FIZZ1* and *Ym1* compared to untreated cells, while LPS being added to untreated cells does not affect the genes’ expression (Figure 1B).

Endotoxin treatment did not modulate the pro-inflammatory markers *IL-1β*, *iNOS*, *TNFα*, decreased *CXCL10* expression, and induced *C3* and *GBP2* expression. There was no influence on the alternative activation-related genes *IL-10, MRC1, FIZZ1*, and *Ym1* (Figure 1). Levels of *iNOS*, *TNFα* and *IL-10* mRNA expression for all treatments were comparable with previously published data [26].

Taken together, the results indicate that long-term treatment by anti-inflammatory interleukins or pro-inflammatory LPS allows cultured astrocytes to exhibit various adaptation states resulting in gene expression profiles related to classical (A1-like) and alternative (A2-like) activation.

### 2.2. The Effect of Adaptations to Anti-Inflammatory Cytokines on an Acute Inflammatory Response

**Figure 2:**
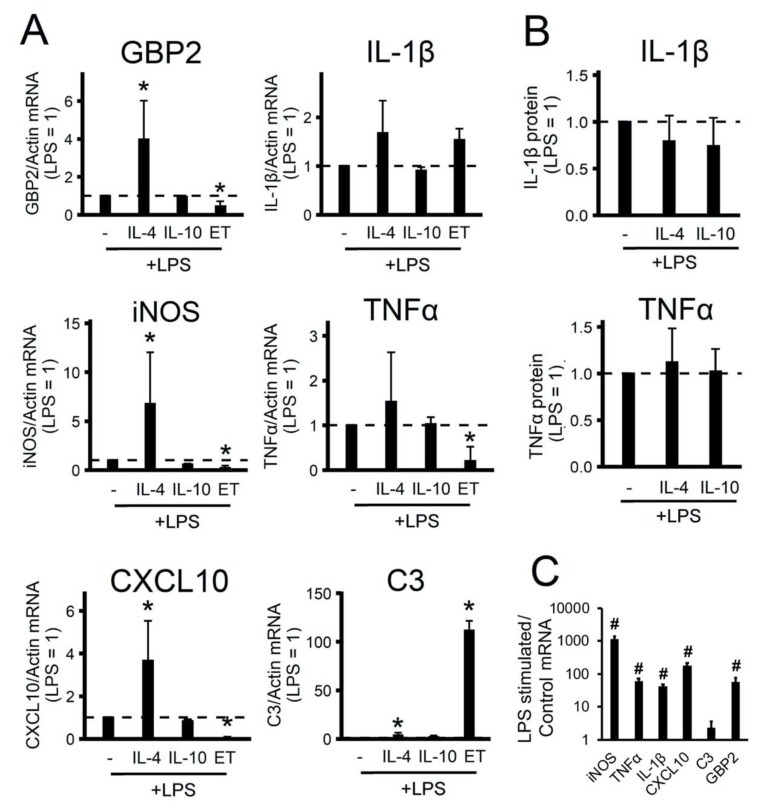
Effects of polarized astrocytes on the inflammatory response. The primary rat astrocyte cultures were pretreated with IL-4 (10 ng/mL) or IL-10 (20 ng/mL) for 24 h or adapted to endotoxin in the tolerance model (LPS 10 ng/mL, 48h, ET) and then stimulated with LPS (100 ng/mL) for 4h (the level of gene expression under LPS stimulation is shown by the dotted line). (**A**), (**C**): the mRNA levels were determined by real-time RT-PCR. The values are normalized to β-actin mRNA levels. (**B**): the TNFα and IL-1β protein release measured by ELISA in supernatant samples. The results are expressed as fold-changes, relative to the LPS-stimulated cells. (**C**): the results are represented as lg, relative to the control cells. The values represent a mean ± SEM from three independent experiments. * *p* < 0.05, compared with the LPS-stimulated cells, # *p* < 0.05, compared with the unstimulated cells.

We found that gene expression profiles allowed the treated astrocytes to be subdivided into two groups that can be attributed to alternative adaptation states (for IL-4, IL-10, and ET treatments) or classical pro-inflammatory stimuli states (for LPS treatment). In the next stage, we examined how the alternative adaptation states differed in the ability of cells to respond to acute LPS stimulation. Lipopolysaccharide was used as an imitator of an innate immune response. The cells were adapted to IL-10 (20 ng/mL) or IL-4 (10 ng/mL) for 24 h, or to a low concentration of LPS (10 ng/mL) for 48 h, then the culture medium was changed and the cells were stimulated with acute LPS (100 ng/mL) for 4 h. The responses were estimated by the expression of pro-inflammatory marker genes (Figure 2A) and the release of IL-1β and TNF protein (Figure 2B).

The acute addition of LPS for 4 h to the naive cells induced the expression of pro-inflammatory markers, except *C3* (Figure 2C), as this gene belongs to the category of slow LPS-activated genes, the expression of which is a notable increase after 9 h of stimulation [30]. We discovered that the IL-4 treatment of cells induced sensitivity to LPS stimulation (increased gene expression *GBP2, iNOS, CXCL10, C3*) (Figure 2A). Notably, IL-10-treated cells had the same responses to LPS as naive cells (Figure 2A). ET treatment reduced the expression of *GBP2 iNOS, TNFα, CXCL10*, but significantly increased *C3* expression (Figure 2). The data show that treatment with IL-4 (but not IL-10) cytokines altered the ability of astrocytes to activate the innate immunity signaling pathways in response to an inflammatory stimulus. Prolonged treatment with low LPS concentrations (ET treatment) makes cells less sensitive to acute LPS stimulation than the IL-4 treatment state.

### 2.3. Oxylipin Profiles of Astrocytes with Different Adaptation States

**Figure 3:**
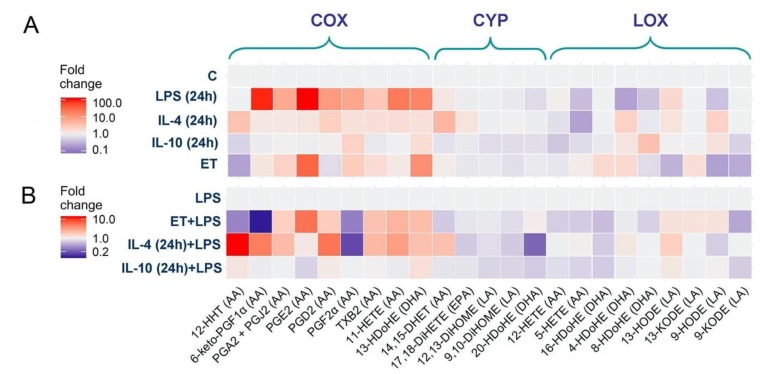
Effect of astrocyte polarization on the oxylipins release. A heat map representation of oxylipin production of n-6 and n-3 fatty acid-derived lipid mediators. (**A**) The primary rat astrocytes were treated with IL-4 (10 ng/mL), IL-10 (20 ng/mL), or LPS (100 ng/mL) for 24 h or adapted to endotoxin in the tolerance model (ET). (**B**) Primary rat astrocytes were pretreated with IL-4 (10 ng/mL), IL-10 (20 ng/mL), or adapted to endotoxin (ET, LPS 10 ng/mL for 48 h) and were then stimulated with LPS (100 ng/mL) for 4 h. Concentrations of oxylipins in supernatants were measured using UPLC-MS/MS. The heat map shows relative amounts of each lipid mediator compared to the control. The horizontal axis indicates the stimuli, while the vertical axis indicates the relative amount (log2) of each lipid mediator. Metabolites were divided into: Lipoxygenase (LOX), cyclooxygenase (COX), and cytochrome (CYP) pathways involved in their synthesis.

Oxylipins are lipid-signaling molecules produced by multiple enzymatic reactions and are derived from the oxidation of PUFAs [5]. The recent development of mass spectrometry has enabled the analysis of oxylipin profiles [5,27]. To characterize whether cultured astrocytes adapted for various cytokines or LPS possess various abilities for oxylipin synthesis, we compared the oxylipin profiles in rat astrocyte cultures following long-term exposure to LPS (100 ng/mL), IL-4 (10 ng/mL), IL-10 (20 ng/mL), and low-concentration LPS (ET, 10 ng/mL). There was a clear difference between the tested phenotypes (Figure 3A). The data are represented as a heat map, with the vertical axis indicating the stimuli and the horizontal axis indicating the relative amount of each lipid mediator (quantitative data presented in Figure S1). 

LPS-treated cells demonstrated a significant increase in the concentration of the eicosanoids (i.e., arachidonic acid (AA) metabolites) 6-keto-PGF_1α_, PGA_2_ + PGJ_2_, PGE_2_, PGD_2_, PGF_2α_, TXB_2_, and 11-HETE, as well as docosahexaenoic acid (DHA) metabolite 13-HDoHE (Figure 3A). There was a decrease in the DHA metabolites 20-HDoHE, 8-HDoHE, 4-HDoHE, and the AA metabolite 5-HETE (Figure 3A). IL-4-treated cells demonstrated other changes in oxylipin profiles. There was a slight increase in 12-HHT, 14,15-DHET, 4-HDoHE, 8-HDoHE, and 9-HODE, as well as a decrease in 5-HETE (AA metabolite) (Figure 3A). IL-10-treated cells revealed no changes in COX-derived metabolites, there were slight changes in LOX-derived metabolites, i.e., an increase in 8-HDOHE, 9-HODE, and a decrease in 12-HETE, 5-HETE (Figure 3A). IL-10-treated astrocytes also synthesized less CYP-derived DHA metabolites 12,13-DiHOME, 9,10-DiHOME, 20-HDoHE. The ET-treated cells (Figure 3A) increased the concentrations of the COX metabolites PGE_2_, PGA_2_+PGJ_2_, and 13-HDoHE, and decreased 12-HHT, 8-HDoHE, 13-HODE, and 9-HODE. Accordingly, an adaptation of the cells to various environmental factors changed their oxylipin profiles.

The difference between the astrocytes’ adaptation states was also found in their sensitivity to acute LPS stimulation (Figure 3B). To estimate the functional responses of alternative adapted cells, we compared low-concentration LPS- (ET), IL-4- and IL-10-treated cells with naive cells in terms of their potential to synthesize oxylipins in response to acute stimulation with LPS (4 h, 100 ng/mL). Oxylipin levels in LPS-stimulated naive cells were taken as 1 (Figure 3B). For ET-treated cells, there was a decrease in PGF_2α_, 6-keto-PGF_1α_, 12-HHT, 14,15-DHET, 12-HETE, and 5-HETE and an increase in PGA_2_+PGJ_2_, PGE_2_, PGD_2_, TXB_2_, 11-HETE, and 13-HDoHE (quantitative data presented in Figure S2). The data coincide with previously published findings [26]. For IL-4-treated cells, there was a decrease in PGF_2α_ 16-HDoHE and 20-HDOHE, and an increase in 12-HHT, PGA_2_+PGJ_2_, PGD_2_, TXB_2_, 11-HETE, 14,15-DHET. For IL-10-treated cells, the LPS-modulation of COX-derived metabolites was not altered in comparison with LPS-stimulated non-treated cells, but a decrease in the DHA metabolites 16-HDOHE, 20-HDOHE was notable (quantitative data presented in Figure S2).

Thus, the data make it possible to conclude that there is a significant difference between the classical and alternatively adapted astrocytes in the synthesis of oxylipin profiles, manifested in the functional properties of these adaptation states, which enables various responses of the cells to acute stimulation with LPS.

## 3. Discussion

Astrocytes play irreplaceable roles in sustaining normal neurological functions and responding to all forms of brain injury and disease [12,22,31]. Following various forms of stimulation, astrocytes undergo rapid changes in gene expression, morphology, and function, collectively referred to as astrocyte reactivity, with brain disturbances associated with abnormal changes in reactivity, i.e., various astrocyte polarization states [12,15,22]. Understanding the mechanisms modulating astrocyte reactivity represents a new direction for identifying potential therapeutic targets for neurological disorders [15,22,31]. These studies raise a question in terms of the characterization of changes in astrocytes during prolonged exposure to substances involved in the responses of innate immunity and associated with neuroinflammation. Such substances are anti-inflammatory cytokines (IL-4, IL-10). In this study, using the polarization gene expression markers proposed earlier [23,28], we demonstrated that rat primary astrocytes can be induced to exhibit pro-inflammatory, A1-like astrocyte reactivity by classical activation with LPS, while alternative activation stimuli, such as IL-4 and IL-10, induce the expression of distinct phenotypic markers in astrocytes. Both IL-4- and IL-10-treated cells more or less can be attributed to A2-like astrocyte reactivity [23], although the differences in these phenotypes were noticeable in the expression of all tested so-called alternative markers: *IL-10, MRC1, FIZZ1,* and *Ym1*. Although there are specificities between astrocytes from various sources (for instance, in glial fibrillary acidic protein (GFAP) or vimentin expressions) [32,33] and there are differences between the expression of some pro- or anti-inflammatory markers in astrocytes obtained from different sources and under various experimental conditions [23,31,34,35], the results indicate similarity in the adaptive responses of astrocytes. Although both IL-4- and IL-10-treated cells can be attributed to A2-like astrocyte reactivity, differences in these phenotypes were noticeable in the expression of all tested so-called alternative markers: *IL-10, MRC1, FIZZ1*, and *Ym1*. It is interesting to note that we observed the effects of pro-inflammatory marker expression during LPS stimulation that were similar to the effects reported in a previous study (23). It is worth noting that the previous work was performed with murine astrocytes and, which might be of more importance, expression was evaluated after 8 h [23] rather than after 24 h, as in the present work. Perhaps the development of an adaptive response by 24 h is associated with a decrease in expression of pro-inflammatory markers *IL-1β, TNF, CXCL10*, which we observed with the addition of IL-4, whereas at 8 h, the effect of IL-4 on the expression of these genes was not observed [23]. Further detailed studies are required to demonstrate whether the observed differences are related to the duration of cell activation. This work shows that, for the proper characterization of the state of astrocytes by marker expression, the conditions for gene expression and their sensitivity to regulation via feedbacks should be taken into account.

We also compared IL-4- and IL-10-treated astrocytes with ET-treated astrocytes. The present markers’ expression data showed similarities between ET- and IL-4-treated states (seven out of 10 genes showed similarities between the two treatments). Nevertheless, the difference in tested genes’ mRNA expression was significant in their sensitivities to acute LPS stimulation. Indeed, we focused on the functional potential of various alternative (A2-like) astrocyte reactivities, measured by their sensitivity to LPS-stimulated acute responses. The IL-10-treated astrocytes revealed the same sensitivity to LPS as naive cells. Surprisingly, we observed an increase in the sensitivity of IL-4-treated astrocytes to the LPS-induced expression of some pro-inflammatory markers (*GBP2, iNOS, CXCL10, C3*). ET treatment resulted in tolerance for acute LPS stimulation, i.e., a decrease in *GBP2, iNOS, TNFα,* and *CXCL10* mRNA expression. Such changes for *TNFα* and *iNOS* were shown previously [26]. The mechanism of these changes and the functional significance of such transcriptional adaptations still need to be investigated further, but such differences in the responses of cells adapted to anti-inflammatory stimuli upon the activation of innate immunity signaling pathways should be taken into account during a study of the mechanisms of inflammatory responses in CNS.

The initial astrocyte separation into A1 and A2 polarization types were proposed based on the neurotoxicity function (31). The search for other functional markers continues. The opposing roles of LPS and IL-4/IL-10 in the functional regulation of astrocytic phenotypes have been documented for such functions as GFAP expression [36], the release of brain-derived neurotrophic factors in astrocytes [37] and the release of glucose [38]. Our data allow us to add oxylipins to this list.

**Figure 4:**
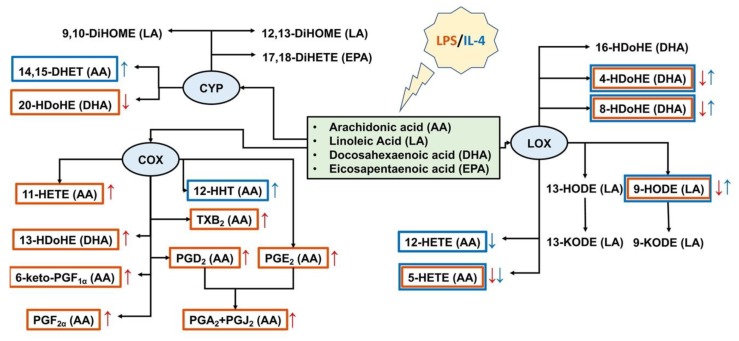
Schema of oxylipins’ profiles for astrocytes classical (LPS) and alternative (IL-4) polarization states. Tested oxylipins, synthesized via lipoxygenase (LOX), cyclooxygenase (COX), or cytochrome (CYP) metabolic branches, are marked with a red frame for classical and a blue frame for alternative polarizations. An arrow of the corresponding color means a decrease (↓) or an increase (↑) in the synthesis of a metabolite.

Other than the release of pro- and anti-inflammatory cytokines, responses to inflammatory stimuli are characterized by oxylipin synthesis [39]. Oxylipins are a class of substances that includes both pro-inflammatory compounds and resolution substances, which are responsible for restoring the system after the pro-inflammatory stimulus has been applied [3,6]. Under pro-inflammatory stimuli, phospholipase A2 cuts PUFAs from the sn-2 position of phospholipids with different lengths of the carbon chain and different numbers and positions of double chemical bonds: C18 (linoleic acid (LA)), C20 (AA, eicosapentaenoic acid (EPA)), C22 (DHA). Oxylipins are formed from PUFAs via the LOX, CYP450, and COX pathways or non-enzymatically [5,39,40]. Oxylipins have multiple effects on cellular responses, including pro- and anti- inflammatory actions via specialized plasma membrane receptors, nuclear receptors, or other mechanisms [39,40]. Although many oxylipins are released in low concentrations, their effects can be summarized [41]. The generalized schema of tested oxylipins and their synthesis pathways via COX-, LOX-, CYP- branches for classical and alternative astrocyte responses are presented in Figure 4. Although some oxylipins can be synthesized enzymatically or non-enzymatically [5], for simplification, we have attributed them to enzymatic branches. We observed that the LPS-stimulated classically polarized cells demonstrated a significant increase in the amounts of AA metabolites of the COX-metabolic branch (Figure 4), which is in line with previous data [16]. In addition, there was an increase in 13-HDoHE, a metabolite of docosahexaenoic acid, via the COX-2 pathway [42]. It is possible to regard the metabolites of the COX pathway as markers of classically polarized phenotypes. It should be noted that there was a simultaneous decrease in a LOX-mediated metabolism that may reflect competition for substrates between various branches of oxylipin metabolism. This requires further clarification. As markers of the alternative polarized state for IL-4, we observed an increase in 14,15-DHET and 9-HODE, 4-HDoHE, 8-HDoHE. 14,15-DHET is a stable metabolite of 14,15-EET, generated from AA by cytochrome P450 epoxygenases [43]. This substance enhances cell viability against oxidant-induced injury [44]. 9-HODE can be synthesized via both enzymatic and non-enzymatic pathways [45] and is considered to be an anti-inflammatory substance [46]. In general, we observe the predominant involvement of the ω-6 PUFA and COX-branch for classical (A1-like) and the ω-3 PUFA and LOX branch for alternative (A2-like) astrocyte responses.

For ET-treatment, there were no notable changes in eicosanoid synthesis besides an increase in the so-called cyclopentenone prostaglandins PGA_2_ and PGJ_2_ (AA-COX pathway). The oxylipin profiles revealed changes in the PUFAs used. A decrease in 9-HODE, 13-HODE (the LA-LOX pathway), and 8-HDoHE (DHA, LOX or non-enzymatically) and an increase in 13-HDoHE (DHA-COX pathway) means that this type of adaptation is closer to classical, A1-like astrocyte responses.

The data allow us to propose that oxylipin profiles are necessary for the characterization of astrocytes’ polarized states. Responses to acute LPS highlight similarities in oxylipin synthesis between classical and alternative phenotypes, i.e., an increase in pro-inflammatory and a decrease in anti-inflammatory oxylipins.

## 4. Materials and Methods

### 4.1. Reagents

Lipopolysaccharide (LPS) (Sigma-Aldrich, cat.no L2630 St. Louis, MO, USA), streptomycin–penicillin (cat.no A063), trypsin (cat.no P037), EDTA, fetal bovine serum (cat.no BS-110/500) were from PanEco (Moscow, Russia). Culture medium Dulbecco’s Modified Eagle Medium (DMEM) (cat.no 21885-025) was sourced from Gibco (Thermo Fisher Scientific, Waltham, MA, USA). Rat IL-4 (400-04, Peprotech), IL-10 (400-19, Peprotech) Oasis^®^ PRIME HLB cartridge (60 mg, 3cc, cat.no. 186008056) were obtained from Waters (Eschborn, Germany).

### 4.2. Primary Cell Culture

**Figure 5:**
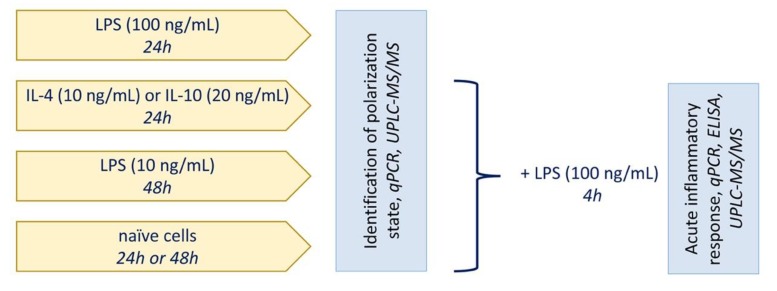
General scheme of treatment.

The cells were obtained from one- or two-day-old pups of Wistar rats. All of the experimental procedures were performed according to the guidelines in the European Convention for the Protection of Vertebrate Animals used for Experimental and Other Scientific Purposes. The cultures of primary rat astrocytes were obtained from newborn rats of both sexes, as previously reported [20]. In brief, the brains from decapitated pups were rinsed with ice-cold Puck’s solution (137.0 mM NaCl, 5.4 mM KCl, 0.44 mM KH2PO4, 0.3 mM Na2HPO4, and 5.5 mM glucose, pH 7.4) and triturated against nylon meshes with the pores of 250 and 136 μm, in a consecutive order. The dissociated cells were plated into 75 cm2 culture flasks at a density of 6 × 105 cells per mL. The cells were subsequently cultured in DMEM (1 g/L D-glucose, 10% bovine fetal serum (FBS), 50 units/mL streptomycin, 50 μg/mL penicillin) at 37 °C, with 10% CO2. After five days of cultivation in DMEM, the culture medium was replaced with a fresh medium and the flasks were placed on a shaker at 200 rpm for 4 h to dissociate the microglial cells. The microglia-containing medium was discarded and the astrocytes-enriched cultures were further grown for the following four days, and the medium was replaced every two days. Subsequently, the cells were washed with phosphate buffered saline, detached from the plastic with trypsin–EDTA solution, plated into six-well plates, and were maintained for two days in DMEM. After this, the medium was replaced by the medium of the same composition, and the cells were used for the experiments. The cells were treated for indicated times with 10 ng/mL IL-4, 20 ng/mL IL-10, and 100 ng/mL or 10 ng/mL LPS. The general scheme of treatment is shown in Figure 5.

### 4.3. Measurement of the Relative RNA Expression Level

We estimated the expression of genes in rat astrocyte cultures: IL-1β (Interleukin 1 beta), C3 (complement component 3), GBP2 (Interferon-induced guanylate-binding protein 2), CXCL10 (C-X-C motif chemokine 10), MRC1 (Mannose receptor C-type 1), FIZZ1 (found in inflammatory zone 1), Ym1 (chitinase-3-like-3), iNOS (inducible nitric oxide synthase), TNFα (Tumor necrosis factor alpha).

Total mRNA was isolated using the GeneJET RNA Purification Kit (Thermo Scientific, Waltham, MA, USA). The concentration of RNA was measured using an Implen NanoPhotometer C. cDNA was generated according to the manufacturer’s instructions using the MMLV RT kit (Evrogen, Moscow, Russia) with oligo-(dT)-primers. Real-time PCR was performed using the 5x PCR-HS-SYBR mix (Evrogen, Moscow, Russia) and the DTlite 4 amplificator (DNATechnology, Moscow, Russia). The sequences of PCR primers used in this study are presented in Table 1.

The annealing temperature was 57 °C. The expression of each gene was measured in 25 µL reactions using cDNA synthesized from 70 ng RNA per reaction well. The relative mRNA expression level was determined by the ΔCT method. The β-actin gene was used as a constitutive gene for normalization. The level of normalized gene expression in control cells or in stimulated cells (specified directly in the text) was taken as one.

### 4.4. UPLC-MS/MS Conditions and Sample Preparation

After the cell experiments, the supernatant was collected and stored at −80 °C for further analysis. The cell-free culture media were taken for the solid-phase lipid extraction (Oasis^®^PRIME HLB cartridge (60 mg, 3cc)). The cartridge was washed with 2 mL of 15% methanol containing 0.1% formic acid, and the lipids were sequentially eluted with 500 μL of anhydrous methanol and 500 μL of acetonitrile. The resulting samples were mixed, concentrated by the evaporation of the solvent under a gentle stream of nitrogen, and were stored at −80 °C. For the identification of lipid mediators, the respective lipid extracts were analyzed using a 8040 series UPLC-MS/MS mass spectrometer (Shimadzu, Japan) in multiple-reaction monitoring mode at a unit mass resolution for both the precursor and product ions [41]. Comprehensive analysis of lipid metabolites hydroxydocosahexaenoic acid (HDoHEs), prostaglandins (PGs), hydroxyeicosatetraenoic acids (HETEs), hydroxyoctadecadienoic (HODEs), and dihydroxyoctadecamonoenoic (DiHOMEs) acids was performed by using a composition of internal standards (tetranor-PGEM-d6 (cat.no. 314840), 6-keto PGF_1α_-d4 (cat.no. 315210), TXB_2_-d4 (cat.no. 319030), PGF_2α_-d4 (cat.no. 316010), PGE_2_-d4 (cat.no. 314010), PGD_2_-d4 (cat.no. 312010), Leukotriene (LT) C_4_-d5 (cat.no. 10006198), LTB_4_-d4 (cat.no. 320110), 5(S)-HETE-d8 (cat.no. 334230), 12(S)-HETE-d8 (cat.no. 334570), 15(S)-HETE-d8 (cat.no. 334720), PAF C16-d4 (cat.no. 10010229), Oleoyl Ethanolamide-d4 (cat.no. 9000552), PGA_2_-d4 (cat.no. 310210) (Cayman Chemical, Ann Arbor, MI, USA) and a commercial software method package for lipid mediators (Lipid Mediator Version 2 software package Shimadzu, Kyoto, Japan) according to the manufacturer’s instructions. The concentration of lipids was normalized to the total protein and was expressed as pg/mg. The total protein was determined by the Bradford assay.

### 4.5. Determination of TNFα and IL-1β by Enzyme-Linked Immunoassay

After the cell experiments, the supernatant was collected and stored at −70 °C for further analysis. The levels of the released TNFα and IL-1β were determined using an enzyme-linked immunoassay commercial kits and a Synergy H4 plate reader (BioTek, Winooski, VT, USA), following the manufacturer’s instructions.

### 4.6. Experimental Data Analysis and Statistics

The data are expressed as a mean ± SEM. The Kruskal–Wallis H-Test (differences between treatments) for multiple comparisons and the Mann–Whitney U Test (two-tailed exact test) for pairwise comparisons, followed by Bonferroni’s post hoc test, were used in order to determine statistical significance. *p* < 0.05 was considered statistically significant. All of the experiments were repeated at least three times.

## 5. Conclusions

In conclusion, the long-term stimulation of astrocytes results in their adaptation, which can be measured by gene expression markers or oxylipins. Treatment with IL-4, but not IL-10, primes the ability of astrocytes to activate the innate immunity signaling pathways in response to LPS, whereas ET-treatment makes cells less sensitive to stimulation. In our view, at present, subdivision into classical and alternative phenotypes simplifies the mechanisms of astrocytes’ innate immune response regulation. The finding that oxylipin profiles associated with different states of polarization can be categorized into a pro-inflammatory or anti-inflammatory phenotype opens an opportunity to manipulate the responses of polarized cells to the activation of innate immunity by using low molecular weight inhibitors of oxylipin synthesis modulators. The observed predominant involvement of ω-6 PUFA and the COX-branch for classical (LPS) pro-inflammatory adaptations and ω-3 PUFA and the LOX-branch for alternative (IL-4) anti-inflammatory adaptations highlights the utility of combined use of COX inhibitors and LOX activators for such regulation.

## Figures and Tables

**Table 1:** DNA sequences of the primers used for RT-PCR.

Gene	Forward	Reverse
*IL-1β*	CACCTCTCAAGCAGAGCACAG	GGGTTCCATGGTGAAGTCAAC
*C3*	AAGCCCAACACCAGCTACATC	ACTTCTGATCCTGGCATTCTTCT
*GBP2*	CTCGACTGTGCATCAGGAAA	TAGGTCTGCACCAGGCTCT
*Cxcl10*	TGCAAGTCTATCCTGTCCGC	ACGGAGCTCTTTTTGACCTTC
*Mrc1*	CAACCAAAGCTGACCAAAGGAAG	TTGCCCATGAGATCTTTCGTGT
*Fizz1*	CAACAGGATGAAGACTGCAACCT	GGGACCATCAGCTAAAGAAG
*Ym1*	TTGCTGGGATGCGGAATAA	AGCTCAGTGTTCCTGTCTTTC
*iNOS*	CCACAATAGTACAATACTACTTGG	ACGAGGTGTTCAGCGTGCTCCACG
*TNFα*	CAAGGAGGAGAAGTTCCCAA	TGATCTGAGTGTGAGGGTCTG
*β-actin*	AGATGACCCAGATCATGTTTGAG	GGCATACAGGGACAACACAG

## Supplementary Materials

The following are available online at https://www.mdpi.com/1422-0067/21/5/1780/s1.

## Abbreviations

AA	Arachidonic acid
COX	Cyclooxygenase
CYP450	Cytochrome P450 monooxygenase
DHA	Docosahexaenoic acid
DiHOME	Dihydroxyoctadecamonoenoic acid
HDoHE	Hydroxydocosahexaenoic acid
HETE	Hydroxyeicosatetraenoic acid
HODE	Hydroxyoctadecadienoic acid
LA	Linoleic acid
LOX	Lipoxygenase
PG	Prostaglandin
PUFAs	Polyunsaturated fatty acids
UPLC-MS/MS	Ultra-performance liquid chromatography-tandem mass spectrometry
