# Supplementary material for: Oxylipin Profiles as Functional Characteristics of Acute Inflammatory Responses in Astrocytes Pre-Treated with IL-4, IL-10, or LPS
Source: Int J Mol Sci. 2020 Mar 5;21(5):1780. doi: 10.3390/ijms21051780 (PMC7084882; doi:10.3390/ijms21051780)
Supplement: Supplementary file 1 [file ijms-21-01780-s001.pdf]

Oxylipin profiles as functional characteristics of acute inflammatory responses in astrocytes pre-treated with IL-4, IL-10 or LPS

Supplementary Information

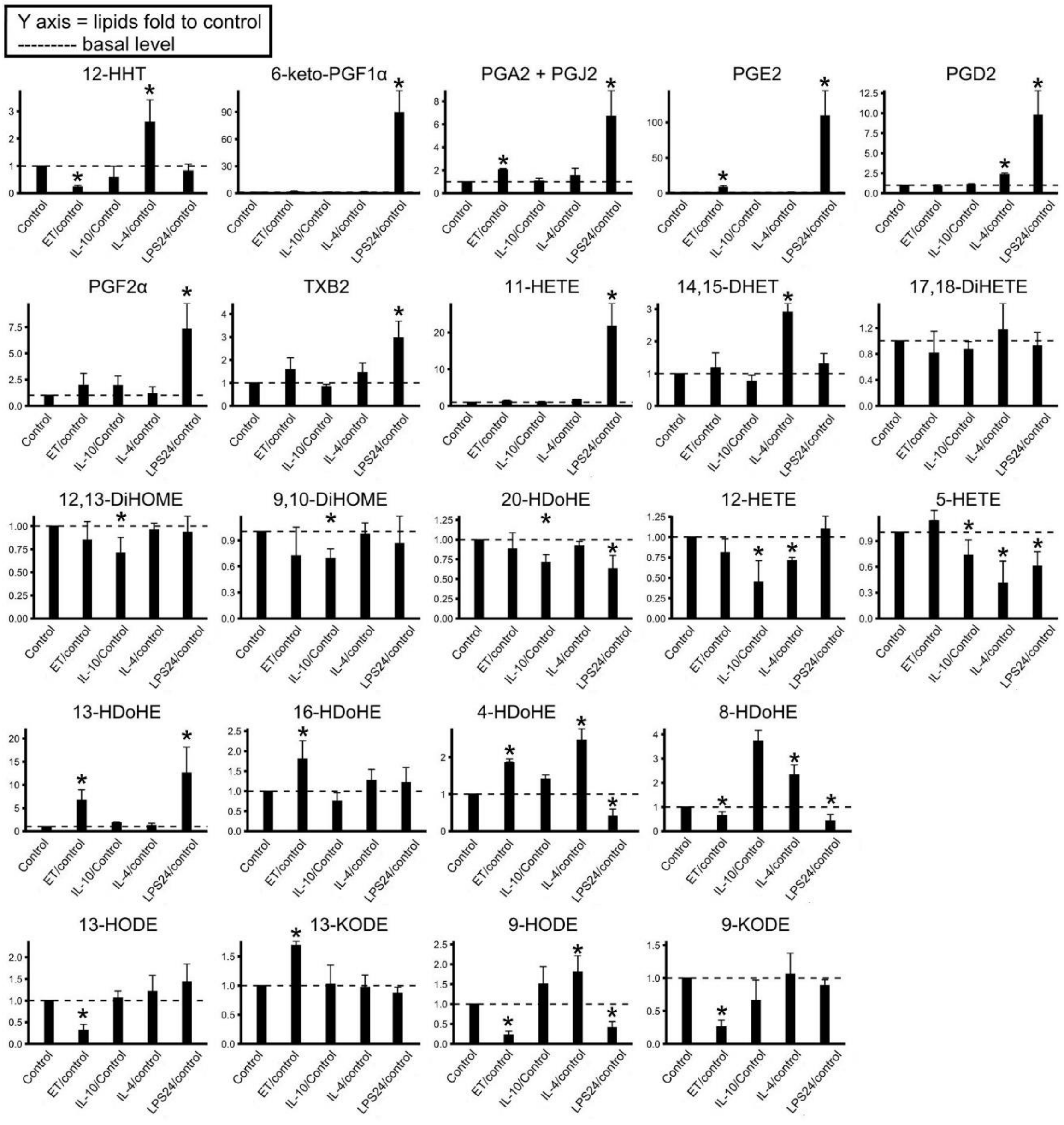

Figure S1. The effect of astrocyte polarization on the oxylipins release. Primary rat astrocytes were adapted to endotoxin in the tolerance model (ET, 10 ng/ml LPS, 48h) or pretreated with IL-10 (20 ng/ml) or IL-4 (10 ng/ml), or LPS (100 ng/ml) for 24 h. Concentrations of oxylipins in supernatants were measured using UPLC-MS/MS. Results are expressed as fold-changes, relative to untreated cells. *$p < 0.05$, compared with the unstimulated cells.

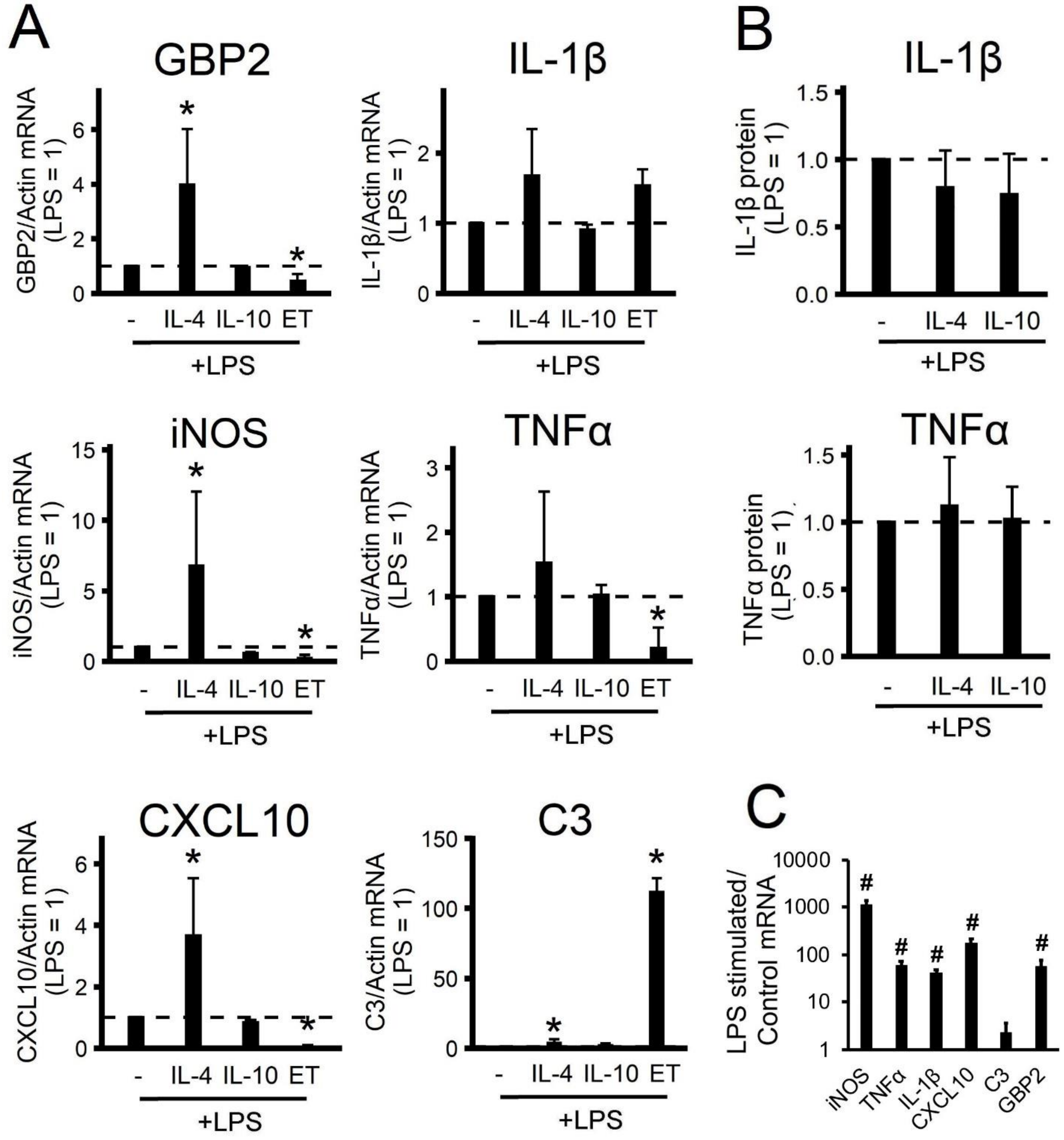

Figure S2. The effect of astrocyte polarization on the oxylipins release. Primary rat astrocytes were adapted to endotoxin in the tolerance model (ET, 10 ng/ml LPS, 48h) or pretreated with IL-10 (20 ng/ml) or IL-4 (10 ng/ml), or LPS (100 ng/ml) for 24 h and then stimulated with LPS (100 ng/ml) for 4 hours. Concentrations of oxylipins in supernatants were measured using UPLC-MS/MS. Results are expressed as fold-changes, relative to LPS-stimulated cells. *$p < 0.05$, compared with the LPS-stimulated cells.
